# Genetic association and functional implications of *TLR4* rs1927914 polymorphism on colon cancer risk

**DOI:** 10.1186/s12885-024-12604-z

**Published:** 2024-07-18

**Authors:** Ang Li, Hui Gao, Hongjiao Wu, Yuning Xie, Zhenxian Jia, Zhenbang Yang, Zhi Zhang, Xuemei Zhang

**Affiliations:** 1 School of Public Health, North China University of Science and Technology, Tangshan, 063210 China; 2 College of Life Science, North China University of Science and Technology, Tangshan, 063210 China; 3 Hebei Key Laboratory of Occupational Health and Safety for Coal Industry, Tangshan, 063210 China; 4 School of Basic Medical Sciences, North China University of Science and Technology, Tangshan, 063210 China; 5 Affliated Tangshan Gongren Hospital, North China University of Science and Technology, Tangshan, 063000 China

**Keywords:** TLRs, Colon cancer, Single nucleotide polymorphism, Cancer susceptibility

## Abstract

**Background:**

Colon cancer remains a major health concern worldwide, with genetic factors playing a crucial role in its development. Toll-like receptors (TLRs) has been implicated in various cancers, but their role in colon cancer is not well understood. This study aims to identify functional polymorphisms in the promoter and 3′UTR regions of *TLRs* and evaluate their association with colon cancer susceptibility.

**Methods:**

We conducted a case-control study involving 410 colon cancer patients and 410 healthy controls from the Chinese population. Genotyping of polymorphisms in *TLR3*,* TLR4*,* TLR5* and *TLR7* was performed using PCR-RFLP and TaqMan MGB probes. Using logistic regression analysis, we evaluated the association of *TLRs* polymorphisms and the susceptibility to colon cancer. To understand the biological implications of the *TLR4* rs1927914 polymorphism, we conducted functional assays, including luciferase reporter assay and electrophoretic mobility shift assay (EMSA).

**Results:**

Our results demonstrated that the G-allele of the *TLR4* rs1927914 polymorphism is significantly associated with a decreased risk of colon cancer (*OR* = 0.68, 95%*CI* = 0.50–0.91). Stratified analysis showed that *TLR4* rs1927914 AG or GG genotype contributed to a decreased risk of colon cancer among younger individuals (*OR* = 0.52, 95%*CI* = 0.34–0.81), males (*OR* = 0.58, 95%*CI* = 0.38–0.87), non-smokers (*OR = 0.58*, 95%*CI =* 0.41–0.83) and non-drinker with *OR* (95%*CI*) of 0.66 (0.46–0.93). Functional assays demonstrated that in HCT116 and LOVO colon cancer cells, the luciferase activity driven by the *TLR4* promoter with the rs1927914A allele was 5.43 and 2.07 times higher, respectively, compared to that driven by the promoter containing the rs1927914G allele. Electrophoretic mobility shift assay (EMSA) results indicated that the rs1927914G allele enhanced transcription factor binding. Using the transcription factor prediction tool, we found that the G allele facilitates binding of the repressive transcription factor *Oct1*, while the A allele does not.

**Conclusion:**

The *TLR4* rs1927914 polymorphism influence the susceptibility to colon cancer, with the G allele offering a protective effect through modulation of gene expression. These insights enhance our understanding of the genetic determinants of colon cancer risk and highlight *TLR4* as a promising target for cancer prevention strategies.

**Supplementary Information:**

The online version contains supplementary material available at 10.1186/s12885-024-12604-z.

## Introduction

Inflammation, a crucial component of innate immunity, is now recognized as a hallmark of cancer development and progression [1]. Chronic inflammation significantly impacts various cancers, including colon cancer [2]. The inflammatory response promotes carcinogenesis through multiple mechanisms, such as the anti-apoptotic effect of nuclear factor-κB (*NF-κB*), DNA oxidative damage, and alterations to the tumor environment [3–5]. Toll like receptors (*TLRs*) are important innate immune molecules that belong to the family of pattern recognition receptors (PRRs) [6]. TLRs induce the release of various cytokines and inflammatory factors by recognizing and binding to pathogen-related molecular patterns (PAMP), thereby participating in the immune inflammatory response [7]. Specifically, *TLR5* on intestinal epithelial cells regulates the composition of intestinal flora and helps prevent inflammation-related diseases [8]. *TLRs* are expressed not only on immune cells, but also on human tumor cells [9, 10].

According to GLOBOCAN 2022, colorectal cancer is the third most common cancer and the second leading cause of cancer-related deaths worldwide [11]. Environmental factors and lifestyle choices, such as smoking and drinking, are associated with an increased risk of colon cancer [12, 13]. However, it remains unclear why colon cancer develops in some individuals but not others, even exposed to the same risk factors. Studies suggest that genetic variants in cancer-related genes could influence an individual’s susceptibility to colon cancer [14–16].

Given the significant role of *TLRs* in cancer development, we conducted a case-control study to investigate the potential functional single nucleotide polymorphisms (SNPs) in *TLRs* and their contribution to colon cancer development.

## Materials and methods

### Study population

This study includes 410 colon cancer patients and 410 healthy controls. Cases were collected from Jan 2008 to Dec 2016 at Tangshan Renmin Hospital and Gongren Hospital of North China University of Science and Technology (NCST) in China with histopathological confirmation. Cancer-free healthy controls were collected from Tangshan area. All participants were genetically unrelated Han Chinese. Informed consent was provided by every individual. Participant provides 2 ml venous blood sample for DNA extraction. This research was supported by the Institutional Review Board of North China University of Science and Technology.

### The selection of *TLRs* genetic variants

Based on the HCB data from NCBI database, we selected variants with a minor allele frequency (MAF) greater than 0.05 in the promoter and 3’ untranslated regions in *TLR3*,* TLR4*,* TLR5 and TLR7* genes. We used the web-based transcription factor prediction tools (Alibaba2.1 and JASPAR) to predict transcription factor binding sites. Additionally, the SNPinfo web server and mirSNP were applicated to predict microRNA binding sites.

### Determination of *TLRs* genotypes

In this study, genotyping was performed on DNA extracted from peripheral blood samples of colon cancer patients and healthy controls to identify germline polymorphisms associated with colon cancer risk. It is critical to emphasize that the DNA used for genotyping was derived from peripheral blood rather than tumor tissue, thus primarily reflecting the germline genetic status rather than the somatic variations that might be present within tumor cells.

The genotypes for *TLR4* rs1927914 and rs7869402 polymorphisms were determined by Polymerase chain reaction (PCR) and restriction fragment length polymorphism (RFLP) analysis. The primers were synthesized by SinoGenoMax (China, Beijing). The primer pairs used were rs1927914F/ rs1927914R (5′-TAG CAT GAG AAA TGA GGA AGT AAG GG-3′/ 5′-GAG CTA TGA TGA GGA TTG AAA ATG TGG-3′) and rs7869402F/ rs7869402R (5′-TGG GAT CCC TCC CCT GTA GC-3′/ 5′-AGG AGC ATT GCC CAA CAG G-3′). PCR was performed in a 6 µl reaction mixtures containing 20ng genomic DNA, 0.1µM each primer and Taq PCR StarMix (GenStar, China). The PCR procedure involved an initial denaturation step at 94 °C for 5 m, followed by 30 cycles of denaturation at 94 °C for 20s, annealing at 59 °C for 30s, and extension at 72 °C for 35s, with a final extension at 72 °C for 5 m. The PCR products for amplifying *TLR4* rs1927914 (524 bp) and rs7869402 (102 bp) were digested by NSi *I* and Alu *I* (NEB, Ipswich, USA), respectively, and the resulting fragments were analyzed by agarose-gel electrophoresis.

To genotype other genetic variants, TaqMan MGB probe-based technique were used. TaqMan SNP genotyping assays (C_27310258, C_8812434 and C_2259573) (Thermo Fisher Scientific, Waltham, USA) were used to genotype *TLR3* rs5743303, *TLR5* rs1640816 and *TLR7* rs3853839, respectively.

To ensure the accuracy and reliability of our genotyping results from PCR-RFLP and TaqMan assays, we implemented a rigorous validation protocol. We randomly selected 10% of the samples from each identified genotype for duplicate testing to assess the consistency and reproducibility of our data. The results from these repeated assays demonstrated perfect consistency, with no discrepancies between the initial and follow-up tests. Additionally, the genotyping results were further confirmed by direct sequencing.

### The construction of *TLR4* luciferase reporter vectors

To confirm the role of *TLR4* promoter variants on transcriptional activity, we prepared allelic reporter constructs containing 1832 bp fragment of the *TLR4* promoter region, spanning from − 1762 bp to 70 bp. The primers used were 5’-GG*G GTA CC*C CGG ATT GGA AGT GCT TGG AG-3’ and 5’-CTA *GCT AGC* TAG AAG AAG AAA ACG CCT GC-3’, which included Kpn *I* and Nhe *I* (NEB, Ipswich, USA) cloning sites (underlined sequences). The PCR product was digested by Kpn *I* and Nhe *I* and then cloned into pGL3-basic luciferase reporter vector (Promega, Madison, USA).

We designed this construct as pGL3-rs1927914A based on the sequence results. Subsequently, we obtained pGL3-rs1927914G plasmids by site-specific mutagenesis using the pGL3-rs1927914 A vector as template. All constructs were verified by direct sequencing.

### Cell culture and transfection for luciferase reporter assay

Colon cancer cell lines (HCT116 and LOVO) were cultured in DMEM medium supplemented with 10% FBS. For each well, 3 × 10^5^ cells were seeded in a 24-well plate and cultured to 80% confluence. The cells were then co-transfected with different pGL3-based constructs and pRL-SV40 using Lipofectamine™ 2000 reagent (Invitrogen, Carlsbad, USA). After transfection, luciferase activities were measured, and the ratio of firefly to Renilla luciferase activities was calculated to determine relative promoter activity. Independent experiments were performed three times.

### Electrophoretic mobility shift assay (EMSA)

Biotin-labeled oligonucleotide probes specific for the rs1927914A (5’-TCT AGG ACT TAG CAT ACA AAT ATT CCT GTT-3’) and the rs1927914G (5’-TCT AGG ACT TAG CAT GCA AA TAT TCC TGT T-3’) variants were synthesized (Sangon Biotech; Shanghai, China). DNA binding ability was assessed using a LightShift™ Chemiluminescent EMSA kit (ThermoFisher Scientific, Waltham, USA). Competitors, identical to the labeled probes but without biotin conjugation, were used to confirm the specificity of the DNA-protein interactions. Nuclear proteins were extracted from HCT116 cells using NE-PER™ Nuclear and Cytoplasmic Extraction Reagents (Thermo Fisher Scientific, Waltham, USA). Nuclear extracts were incubated with 20fmol labeled oligonucleotide probes for 15 min. For competition experiment, 4pmol of unlabeled oligonucleotide competitors were added prior to incubation with the labeled probes. The binding reactions were then subjected to electrophoresis in a 6.5% polyacrylamide gel. The reactions were then transferred to positively charged nylon membrane and examined for chemiluminescence.

### Statistical analysis

The differences in basic demographic information between the healthy population and colon cancer patients were examined using the *χ*^*2*^ test. Hardy-Weinberg Equilibrium of all tested SNPs among controls were also estimated by the *χ*^*2*^ test. The associations of genetic variants in *TLR* genes with colon cancer risk were evaluated by calculating the odds ratios (OR) with 95% confidence intervals (CI), adjusting for possible confounding factors.

Current smokers were defined as those who had smoked up to one year prior to diagnosis (for colon cancer patients) or up to the date of the interview (for controls). Smoking dose was indicated by pack-years, and smokers were categorized into light and heavy smokers based on the median of pack-years value in controls. Differences in luciferase activity were determined by the *t* test. Data analyses were performed using SPSS 23.0 (SPSS, Chicago, USA).

## Results

### Participant information

The basic information for all participants were summarized in Table 1. The median age for both groups was 60 years old. There were no significant differences in the distribution of gender and age between patients and health controls. The distributions of smoking status and drinking status did not differ significantly between the two groups (*P* > 0.05).

**Table 1:** Distributions of selected characteristics of patients with colon cancer cases and controls

Variables	Cases (*N* = 410)	Controls (*N* = 410)	*P*^a^ value
Gender			0.833
Male	225 (54.9%)	228 (55.6%)	
Female	185 (45.1%)	182 (44.3%)	
Age			0.364
≤ 60	198 (48.3%)	211 (51.5%)	
>60	212 (51.7%)	199 (48.5%)	
Smoking status			0.682
Non-smoker	315 (76.8%)	310 (75.6%)	
Smoker	95 (23.2%)	100 (24.4%)	
Drinking Status			0.740
Non-Drinker	318 (77.6%)	314 (76.6%)	
Drinker	92 (22.4%)	96 (23.4%)	
Pack-year smoked			0.596
<30	42 (44.2%)	48 (48.0%)	
≥ 30	53 (55.8%)	52 (52.0%)	

^a^: Two-sided *χ*^*2*^ test

### Association of *TLRs* polymorphisms with colon cancer risk

Bioinformatics analysis predicted that 5 SNPs in the promoter and 3’UTR regions of *TLRs* could affect transcription factor or microRNA binding. For SNPs with a limited number of complete mutations, we combined genotypes with at least one mutant allele for further analysis. The genotype frequencies for each SNP and their association with the risk of colon cancer were presented in Table 2.

**Table 2:** Genotype frequencies of toll-like receptor family gene and their association with Colon cancer

Genotypes	Cases (*N* = 410)	Controls (*N* = 410)	OR (95%CI)^a^	*P* value
*TLR3* rs5743303				
AA	292 (71.2%)	296 (72.2%)		
AT	105 (25.6%)	102 (24.9%)	1.04 (0.76–1.43)	0.818
TT	13 (3.2%)	12 (2.9%)	1.10 (0.49–2.46)	0.814
AT + TT	118 (28.8%)	114 (27.8%)	1.05 (0.77–1.42)	0.778
*TLR4* rs7869402				
CC	359 (87.6%)	358 (87.3%)		
CT	49 (11.9%)	50 (12.2%)	0.97 (0.64–1.48)	0.902
TT	2 (0.5%)	2 (0.5%)	1.02 (0.14–7.30)	0.985
CT + TT	51 (12.4%)	52 (12.7%)	0.98 (0.65–1.48)	0.906
*TLR4* rs1927914				
AA	159 (38.8%)	125 (30.5%)		
AG	194 (47.3%)	214 (52.2%)	0.70 (0.51–0.95)	**0.022**
GG	57 (13.9%)	71 (17.3%)	0.62 (0.40–0.95)	**0.030**
AG + GG	251 (61.2%)	285 (69.5%)	0.68 (0.50–0.91)	**0.010**
*TLR5* rs1640816				
GG	320 (78.1%)	332 (81.0%)		
AG	87 (21.2%)	76 (18.5%)	1.19 (0.84–1.68)	0.321
AA	3 (0.7%)	2 (0.5%)	1.53 (0.25–9.29)	0.646
AG + AA	90 (21.9%)	78 (19.0%)	1.20 (0.85–1.69)	0.294
*TLR7* rs3853839				
Female				
GG	111 (27.1%)	115 (28.3%)		
GC	59 (14.4%)	60 (14.6%)	1.03 (0.66–1.60)	0.911
CC	15 (3.7%)	7 (1.5%)	2.49 (0.93–6.68)	0.070
GC + CC	74 (18.1)	67 (16.1%)	1.16 (0.76–1.78)	0.488
Male				
G	170 (41.4%)	181 (44.9%)		
C	55 (13.4%)	47 (10.7%)	1.41 (0.90–2.22)	0.136

^a^: Data were calculated by logistic regression and adjusted for sex, age(categories), and smoking status, drinking status

Using a multivariate logistic regression model, we found that the genotype frequencies of *TLR4* rs1927914 among cases were significantly different from those among controls. The presence of G-allele in *TLR4* rs1927914 was associated with a decreased colon cancer risk (*OR* = 0.68, 95%*CI* = 0.50–0.91). This multivariate logistic regression model passed the significance test, with Hosmer-Lemeshow test showing a *P* value greater than 0.05 and a predicted accuracy exceeding 50%. Cox-Snell’s *R*^*2*^ and Nagelkerke’s *R*^*2*^ were 0.16 and 0.22, respectively. Additionally, the Omnibus test of model coefficients indicated that the model is effective and has good fit (*P* < 0.05).

Our data didn’t show any association of other *TLRs* SNPs with the susceptibility to colon cancer.

### Stratification analysis of the *TLRs* variants and colon cancer risk

The stratified analysis results were presented in Table 3. The *TLR4* rs1927914G allele containing genotype is associated with a reduced risk of colon cancer in males (*OR* = 0.58, *95%CI* = 0.38–0.87) when stratified by gender. When stratified by age, individuals with at least one rs1927914G allele had a lower risk of colon cancer in younger participants (*OR* = 0.52, *95%CI* = 0.34–0.81), but not in elder participants (*OR* = 0.87, *95%C*I = 0.58–1.31). In analyses stratified by smoking status or drinking status, the *TLR4* rs1927914G containing genotype was found to be a protective factor among non-smokers (*OR = 0.58*, 95%*CI* = 0.41–0.83) and among non-drinkers (*OR* = 0.66, 95%*CI* = 0.46–0.93), but not among smokers or drinkers.

**Table 3:** Stratified analysis between genotypes of *TLR4* rs1927914 and colon cancer risk

Variables	Cases/Controls		Dominant model (AG + GG)/AA OR (95% CI)^a^	*P* value
	AA	AG + GG		
Gender				
Male	91/67	134/161	0.58 (0.38–0.87)	0.009
Female	68/58	117/124	0.79 (0.51–1.22)	0.295
Age				
≤ 60	81/57	127/154	0.52 (0.34–0.81)	0.004
>60	78/68	134/131	0.87 (0.58–1.31)	0.515
Smoking status				
Smoker	49/51	46/49	0.98 (0.56–1.71)	0.936
Non-smoker	110/74	205/236	0.58 (0.41–0.83)	0.002
Drinking Status				
Drinker	46/37	46/59	0.64 (0.35–1.16)	0.143
Non-Drinker	113/88	205/226	0.66 (0.46–0.93)	0.018

^a^: Data were calculated by unconditional logistic regression and adjusted for gender, age, smoking status, and drinking status, where it was appropriate

### Effect of *TLR4* rs1927914 polymorphism on transcriptional activity

**Fig. 1:**
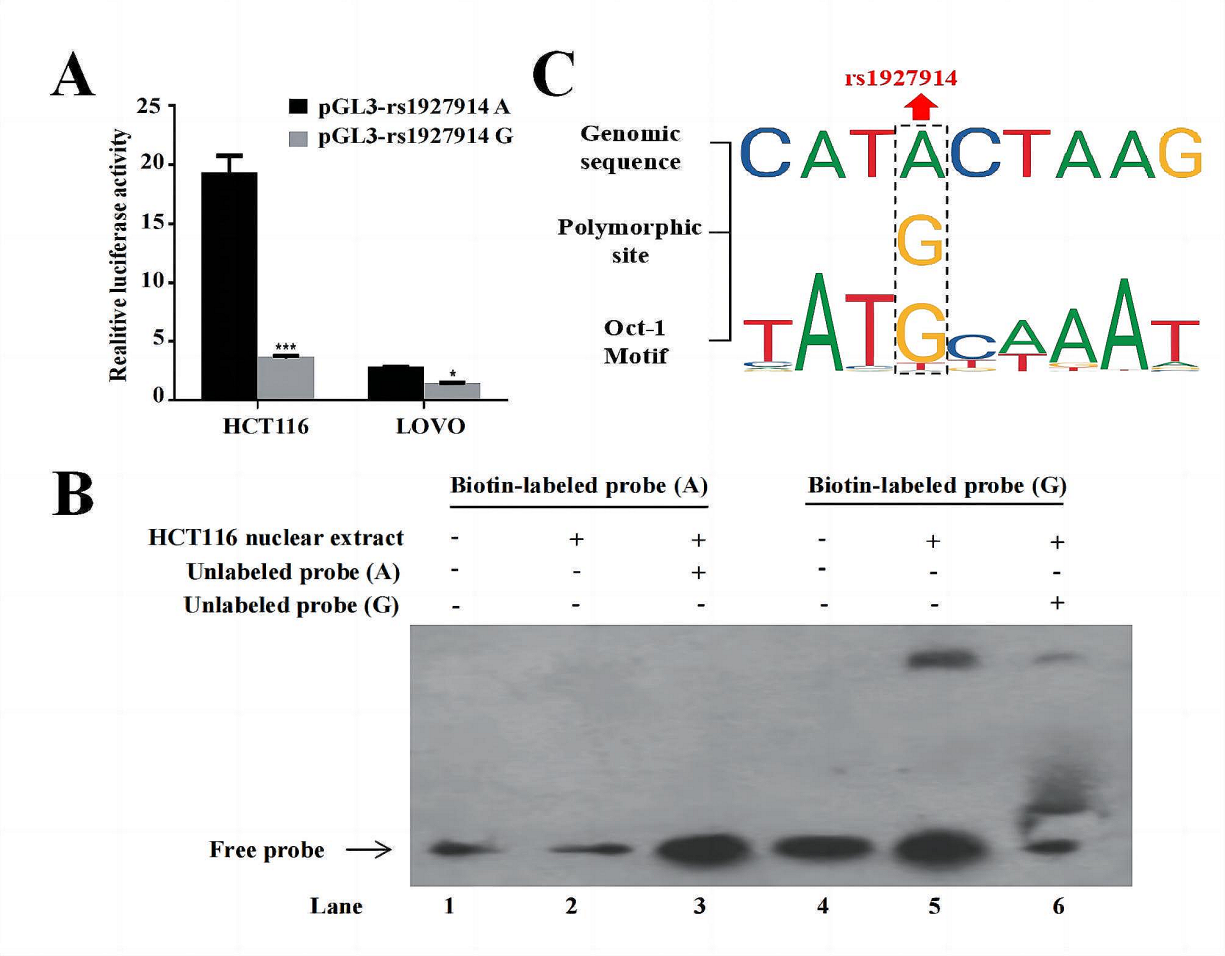
The functional analysis of *TLR4* rs1927914 polymorphism. (**A**) Luciferase expression of two constructers (pGL3-rs1927914G and pGL3-rs1927914A) in HCT116 and LOVO cells co-transfected with pRL-SV40 to standardize transfection efficiency. Fold increase was measured by setting the activity of the empty pGL3-Basic vector as 1. *P < 0.05 **P < 0.01 compared with each of the construct counterparts. (**B**) Electrophoretic mobility shift assays with biotin-labeled oligonucleotide probes containing *TLR4* rs1927914 A or G allele. Nuclear extracts were incubated with 5’-Biotin-TCTAGGACTTAGCAT*A*CAAATATTCCTGTT-3’ (A probe, lanes 1–3) or 5’-Biotin-TCTAGGACTTAGCAT*G*CAAATATTCCTGTT (G probe, lanes 4–6). Lanes 1 and 4 show the gel mobilities of the biotin-labeled probes without nuclear extracts; lanes 2 and 5 show the mobilities of the biotin-labeled probes with nuclear extracts in the absence of unlabeled probes. The binding specificity was confirmed by competing the biotin-labeled A or G probe with a 100-fold molar excess of unlabeled A (lane 3) or G probe (lane 6). (**C**) Transcription factor prediction using JASPAR and AliBaba showed that rs1927914 was consistent with the binding sequence of *Oct-1*

We conducted a luciferase reporter assay to investigate the effect of the *TLR4* rs1927914 polymorphism on transcriptional activity. Constructor pGL3-rs1927914A or pGL3-rs1927914G were co-transfected with pRLSV40 control plasmid in HCT116 and LOVO cells. As showed in Fig. 1A, the results showed that he luciferase activity driven by rs1927914A-containing *TLR4* promote was 5.43 times higher in HCT116 cells and 2.07 times higher in LOVO cells compared to the rs1927914G-containing *TLR4* promoter. These results indicated that the *TLR4* rs1927914G allele was associated with a strikingly lower promoter activity, suggesting a functional impact of this variant on *TLR4* expression.

Given that allele-specific activity of genetic variants in regulatory regions can influence the binding affinity of transcription factors (TFs), we further conducted an EMSA to investigate if different *TLR4* rs1927914 alleles effect the binding activity of transcriptional factor (Fig. 1B). Nuclear extracts prepared from HCT116 cells were incubated with biotin-labeled oligonucleotides for the rs1927914 locus containing either the A or G allele. As shown in the lane 5, shifted bands were observed when the nuclear extract was incubated with the biotin-labeled probe containing the rs1927914G allele, indicating a protein-DNA interaction. In contrast, no shifted bands were observed with the biotin-labeled probe containing the rs1927914A allele (lane 2), suggesting a lack of binding. To confirm the specificity of the binding complex, a 100-fold molar excess of unlabeled oligonucleotide probe was added to the reaction. This competition assay inhibited the formation of the binding complex, as seen in lane 6, further confirming the specificity of the interaction between the nuclear extract and the rs1927914G allele probe. These results demonstrated that the rs1927914G allele had a stronger binding affinity to nuclear protein compared to the A allele. This suggests that *TLR4* rs1927914G allele likely increases the binding of transcription factor, subsequently leading to a significant decrease in *TLR4* promoter activity.

Our electrophoretic mobility shift assay (EMSA) findings prompted us to further explore the impact of the *TLR4* rs1927914 genetic variation on transcription factor binding using JASPAR and Alibaba transcription factor prediction website. The resluts indicated that the *TLR4* promoter sequence with the rs1927914G allele can bind to *Oct1*, whereas that with the rs1927914 A allele cannot (Fig. 1C).

These results suggest that the rs1927914G allele enhances repressive transcription factor *Oct1* binding to the *TLR4* promoter, leading to decreased transcriptional activity of the *TLR4* gene. This mechanism likely explains the reduced *TLR4* promoter activity observed with the G allele in our luciferase reporter assays. Therefore, the presence of the rs1927914G allele in the *TLR4* promoter region may result in lower *TLR4* expression levels due to the increased binding of the repressive transcription factor *Oct1*.

## Discussion

*TLRs* are crucial components of the inflammatory response, impacting the innate immune response. In recent years, numerous studies have elucidated the roles and molecular mechanisms of *TLRs* in the development of various cancers [17, 18]. Cancer susceptibility is influenced by SNPs in these *TLRs*, which can serve as potential biomarker for assessing cancer risk. Genetic variants in *TLRs* may alter ligand binding capability and subsequently modulate cancer risk [19, 20]. Studies has indicated that the effect of *TLR4* SNPs on susceptibility to various cancer types is through the disruption of *TLR4* signaling [21].

Our study investigated the association between specific *TLR* polymorphisms and colon cancer risk. Firstly, we found no significant association between colon cancer risk and the *TLR3* rs5743303, *TLR5* rs1640816, and *TLR7* rs3853839 polymorphisms. These variants are rarely reported in relation to various cancers. Notably, *TLR7* rs3853839 has been identified in one study as a predictive marker for cetuximab-based chemotherapy in colon cancer patients [22], highlighting its potential clinical relevance in treatment response. For *TLR4* rs7869402 polymorphism, it has been reported to reduce the susceptibility to non-small cell lung cancer (NSCLC), small cell lung cancer (SCLC), and gastric cancer [23–25], suggesting a protective role in these cancers. These findings imply that the rs7869402 variant may contribute to lower incidence rates of these cancer types. However, our data did not show any significant influence of *TLR4* rs7869402 on colon cancer risk, indicating that the protective effect of this polymorphism may be cancer-type specific. Additionally, it is worth mentioning that the *TLR4* rs7869402 has been associated with reduced overall survival in ovarian cancer patients [26]. This discrepancy underscores the complexity of genetic influences on cancer susceptibility and prognosis, suggesting that the same genetic variant have different impacts depending on the cancer type and possibly other interacting genetic and environmental factors.

Our study indicated that *TLR4* rs1927914 polymorphism is associated a decreased colon cancer risk, consistent with findings in other cancers. Case-control studies have shown that the rs1927914 polymorphism is significantly linked to a lower risk of prostate cancer, hepatocellular carcinoma, esophageal squamous cell carcinoma, and small cell lung cancer [24, 27–29]. These findings suggest a protective role for the *TLR4* rs1927914 polymorphism across various cancer types. However, some reports indicate no association between *TLR4* rs1927914 and gastric cancer [25]. These discrepancies suggest that the impact of the *TLR4* rs1927914 polymorphism may be context-dependent, influenced by factors such as tissue-specific expression, environmental interactions. The context-dependent nature of these genetic effects highlights the complexity of genetic influences on cancer susceptibility, which may vary by cancer type and population.

Our functional assays provide further insights into the molecular mechanisms underlying these associations. In colon cancer cells, the luciferase activity driven by the *TLR4* promoter containing the rs1927914A allele was significantly higher compared to that driven by the promoter with the G allele. Kutikhin et al. found that high *TLR4* expression in cancer tissues promoted tumor cell metastasis and invasion [30]. In addition, studies have shown that *TLR4* has the potential to become a marker for disease progression in patients with colon cancer [31], and its low expression is correlated with a better prognosis [32]. This suggests that the A allele enhances *TLR4* transcriptional activity, potentially facilitating cancer progression. Conversely, the G allele appears to reduce promoter activity, aligning with its protective role against colon cancer.

Our findings indicate that the rs1927914 G allele enhances the binding affinity of transcription factors, specifically facilitating the binding of the repressive transcription factor *Oct1*, unlike the A allele. This finding aligns with previous studies demonstrating *Oct1’s* role in transcriptional repression. The interaction between *SMRT* and the POU domain of *Oct-1* further supports the mechanistic basis of our observations [33]. In gastric cancer cells, *Oct1* is recruited to the *CDX2* promoter but loses its ability to activate transcription, highlighting its complex regulatory role [34, 35]. Additionally, *Oct-1* inhibits *Slc7a11* and *CRP* gene expression by binding to their promoters [36, 37]. These insights suggest that the protective effect of the *TLR4* rs1927914G allele in colon cancer may result from enhanced *Oct-1* binding, leading to reduced *TLR4* expression and decreased cancer risk. The context-dependent effects of *Oct-1* in different cancer types underscore the complexity of its role in gene regulation and cancer biology.

This study has significant clinical implications for understanding and potentially managing colon cancer risk, particularly in the context of genetic predisposition. The identification of the *TLR4* rs1927914 polymorphism as a factor associated with decreased colon cancer risk provides valuable insights into the genetic underpinnings of this disease. Additionally, understanding the role of *TLR4* in colon cancer progression opens new avenues for therapeutic interventions. Given that high *TLR4* expression is associated with increased metastasis and invasion of tumor cells [32, 38], targeting *TLR4* signaling pathways could be a viable strategy for treatment. The identification of the rs1927914 polymorphism’s impact on *TLR4* promoter activity further supports the potential of *TLR4* as a therapeutic target. However, further research and clinical trials are warranted to translate these findings into practical applications in the clinical setting.

Despite the strengths of our study, several limitations should be acknowledged. One significant limitation is the potential for detection biases inherent in the PCR-RFLP and TaqMan assays. Although we performed duplicate testing and direct sequencing validation on a subset of samples to mitigate this issue, it is possible that some biases or errors could still affect our results. Future studies should consider using multiple genotyping methods or more advanced technologies to further minimize this risk. Additionaly, the study population was limited to the Chinese population, which may affect the generalizability of our findings to other ethnic groups. Genetic variations and their impacts on disease risk can vary significantly across different populations. Lastly, the sample size, while sufficient to detect significant associations, may limit the power to identify more subtle genetic effects or interactions with environmental factors. Larger studies with increased statistical power are needed to uncover additional risk factors and their interactions.

## Conclusions

The *TLR4* rs1927914 polymorphism influence the susceptibility to colon cancer, with the G allele offering a protective effect through modulation of gene expression. These insights enhance our understanding of the genetic determinants of colon cancer risk and highlight *TLR4* as a promising target for cancer prevention strategies.

### Electronic supplementary material

Below is the link to the electronic supplementary material.

Supplementary Material 1

## Data Availability

The datasets used during the current study are available from the corresponding author on reasonable request.

## Abbreviations

TLRs: Toll-like receptors
ORs: Odds ratios
CIs: Confidence intervals
SNPs: Single nucleotide polymorphisms
EMSA: Electrophoretic mobility shift assay
PRRs: Pattern recognition receptors
PAMP: Pathogen-related molecular patterns
