# Supplementary material for: Genetic association and functional implications of TLR4 rs1927914 polymorphism on colon cancer risk
Source: BMC Cancer. 2024 Jul 18;24:858. doi: 10.1186/s12885-024-12604-z (PMC11256370; doi:10.1186/s12885-024-12604-z)
Supplement: Supplementary file 1 — Supplementary Material 1 [file 12885_2024_12604_MOESM1_ESM.pdf]

Supplementary Information

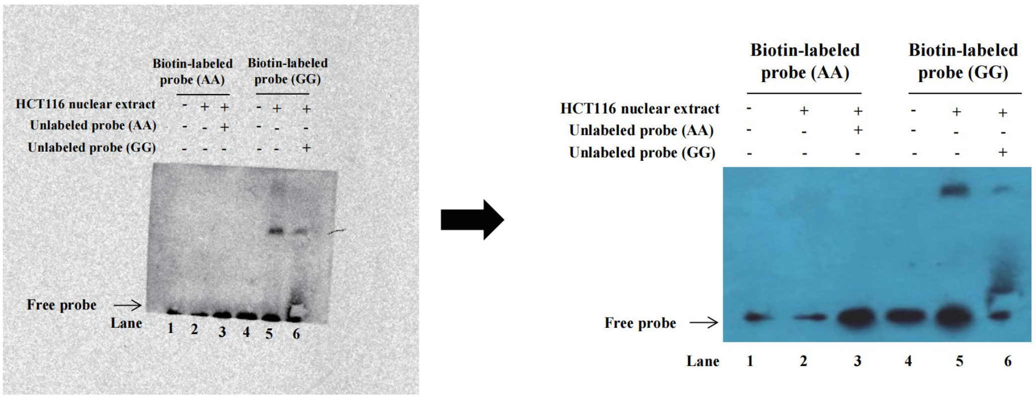

Figure S1 The original EMSA blot image (left) and the corresponding film (right) of the entire membrane. Electrophoretic mobility shift assays with biotin-labeled oligonucleotide probes containing TLR4 rs1927914 A or G allele. The nuclear protein was extracted from HCT116 cells. Eight micrograms of extract were incubated with a biotin-labeled probes TCTAGGACTTAGCATACAAATATTCCTGTT (AA probes, lanes 1 through 3) or TCTAGGACTTAGCATGCAAATATTCCTGTT (GG probes, lanes 4 through 6) in the rs1927914 site. Lanes 1 and 4 show the gel mobilities of the biotin-labeled probes without nuclear extracts; lanes 2 and 5 show the mobilities of the biotin-labeled probes with nuclear extracts in the absence of unlabeled probes; and binding specificity was confirmed by chasing biotin-labeled AA or GG probes with a 100-fold molar excess of unlabeled AA (lane 3) or GG probes (lane 6).
